# Non-occlusive Acute Mesenteric Ischemia in an Adult Male Patient With Diabetic Ketoacidosis

**DOI:** 10.7759/cureus.91707

**Published:** 2025-09-06

**Authors:** Khalid Ahmad, Abdul Hanan Farooq, Ayesha Muneer, Ahmed Gohar, Mario Gonzalez

**Affiliations:** 1 Medicine, Khyber Medical University, Peshawar, PAK; 2 Acute Medicine, University Hospitals Bristol and Weston NHS Foundation Trust, Weston-super-Mare, GBR; 3 Gastroenterology, Salford Royal NHS Foundation Trust, Salford, GBR; 4 Critical Care, North Alabama Medical Center, Florence, USA; 5 Internal Medicine, North Alabama Medical Center, Florence, USA

**Keywords:** diabetic ketoacidosis (dka), exploratory laparotomy, hypovolemia, intestinal ischemia, non-occlusive mesenteric ischemia

## Abstract

Diabetic ketoacidosis (DKA) is a medical emergency that may represent the first manifestation of diabetes mellitus. Management typically centers on fluid resuscitation, insulin therapy, and treatment of precipitating factors, but clinicians must remain alert to rare and life-threatening complications. We report a 28-year-old male with no prior history of diabetes who presented with influenza-associated DKA. Despite standard therapy, he developed worsening shock and persistent abdominal tenderness. Computed tomography of the abdomen revealed portal venous gas and pneumatosis intestinalis, prompting emergent laparotomy. Intraoperative findings confirmed extensive ischemic necrosis of the terminal ileum, right colon, and appendix, requiring resection with temporary abdominal closure. A second-look procedure 48 hours later demonstrated viable remaining bowel, and an ileo-transverse anastomosis with definitive closure was performed. The patient improved steadily, was extubated, and discharged home in stable condition. This case highlights the need for vigilance when abdominal pain or hemodynamic instability persists in DKA patients despite appropriate management. Non-occlusive mesenteric ischemia (NOMI), although rare and more often described in older individuals with vascular risk factors, can occur in young adults in the setting of profound hypovolemia and shock. Early recognition, aggressive fluid resuscitation, careful titration of vasopressors, and timely surgical intervention are critical to improving outcomes and preventing mortality in these patients.

## Introduction

Non-occlusive mesenteric ischemia (NOMI) is estimated to constitute up to 15% of mesenteric ischemia cases [1]. Hypoperfusion of the intestines and failure to meet metabolic demands can lead to ischemia [2]. NOMI is a rare complication of diabetic ketoacidosis (DKA) in the adult population and can be associated with abdominal pain, bowel necrosis, and sudden hypovolemia [3].

DKA most commonly occurs in patients with known diabetes but can occasionally present as the first manifestation of diabetes mellitus. Secondary diabetes, especially due to pancreatic injury such as alcohol-induced pancreatitis, can predispose individuals to significant glucose dysregulation. Infections like influenza often precipitate DKA by increasing metabolic demands and insulin resistance. Recognizing these underlying factors is crucial for diagnosis and management.

We present the case of a young adult male with no known history of diabetes mellitus who presented with DKA complicated by shock and NOMI, highlighting the importance of considering secondary diabetes and infectious triggers in similar clinical scenarios.

## Case presentation

A 28-year-old male with a past medical history of alcohol use disorder and resulting pancreatitis was brought to the emergency department with a chief complaint of altered mental status, noted after a brief syncopal episode the morning of admission. Per the patient's wife, the patient developed flu-like symptoms and vomiting for two days before admission. He self-medicated with an over-the-counter combination of acetaminophen, dextromethorphan, and phenylephrine HCl. In the emergency department, the patient was hypotensive with a blood pressure of 75/40 mmHg. He received a 30 mL/kg crystalloid bolus and was started on a norepinephrine drip. On initial physical examination, the patient was drowsy and confused but followed some commands. His skin was pale and dry, and his abdomen was mildly tender.

**Figure 1:**
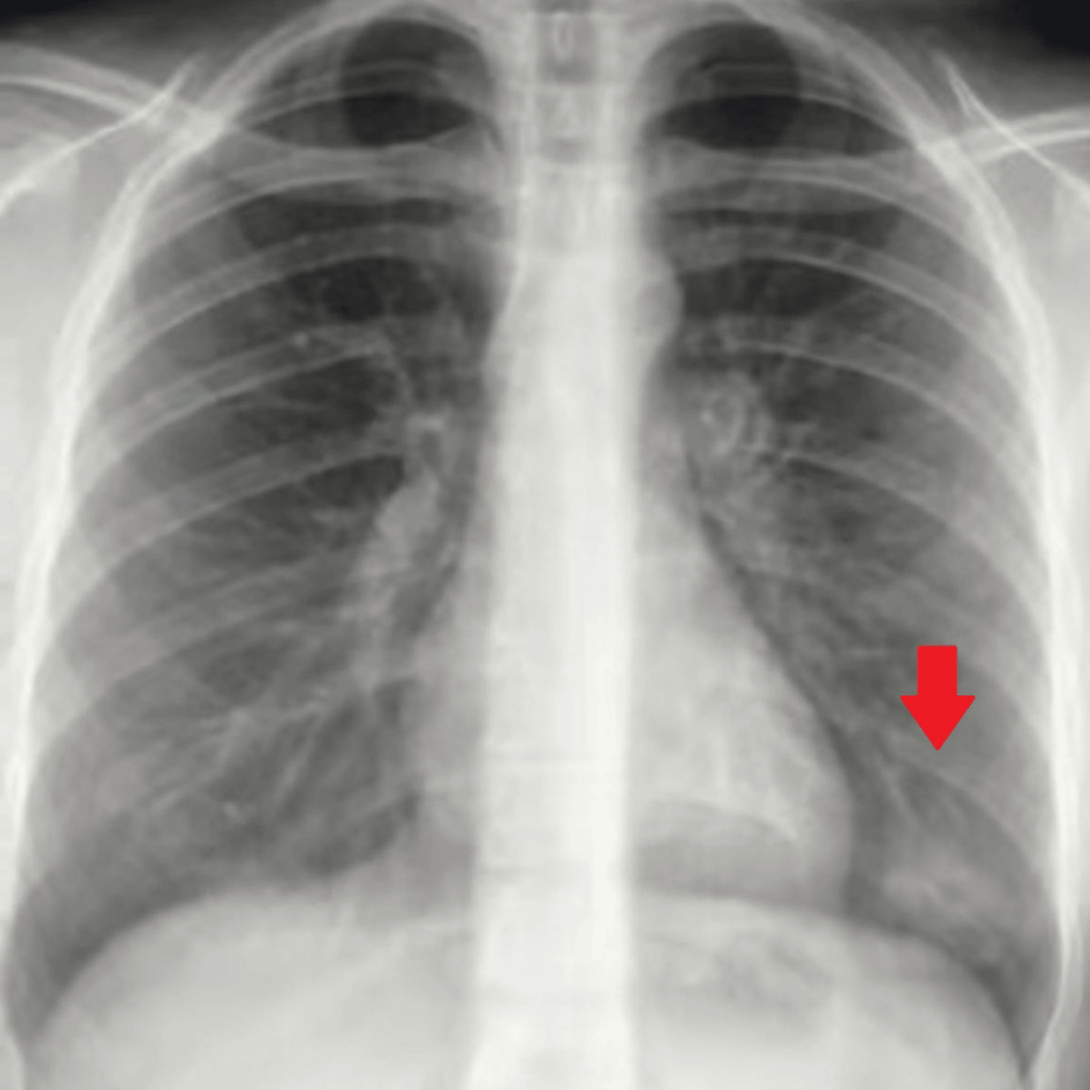
Chest X-ray demonstrating left lower lobe opacity consistent with pneumonia.

Workup (Table 1) was remarkable for positive Influenza type A serology, and a chest X-ray that showed left lower lobe opacity, suggestive of pneumonia (Figure 1). Basic metabolic panel and blood gas analysis reported a pH of 6.91, bicarbonate of 5, an elevated anion gap, potassium of 3.5 mmol/L, and creatinine of 3.2 mg/dL with blood urea nitrogen of 72 mg/dL; beta-hydroxybutyrate was elevated at 10.70 mmol/L, and lipase level was 1210 U/L. The initial X-ray of the abdomen was normal.

**Table 1:** Laboratory findings on initial presentation.

Test	Patient value	Reference range
Blood picture		
White blood cell count (WBC)	15.4 ×10³/µL	4.3–11
Hemoglobin (Hgb)	14.7 g/dL	14–18
Hematocrit (Hct)	47.4 %	40–54
Platelets	141 ×10³/µL	150–375
Blood chemistry		
Sodium	118 mmol/L	135–145
Potassium	3.5 mmol/L	3.6–5.2
Glucose	1189 mg/dL	65–99
HbA1c	18.4 %	2.3–5.6
Lipase	1210 U/L	23–300
Blood urea nitrogen (BUN)	72 mg/dL	4–22
Creatinine	3.2 mg/dL	0.6–1.3
Urinalysis findings		
Urine glucose	>500 mg/dL	Negative
Urine ketones	20 mg/dL	Negative
Arterial blood gases		
pH	6.91	7.35–7.45
pCO₂	19.3 mmHg	35–45
pO₂	92.0 mmHg	75–100
HCO₃	3.9 mEq/L	21–28
Other		
Influenza A	Positive	Negative

The patient was started on oseltamivir, antibiotics, intravenous lactated Ringer's, and intravenous regular insulin drip per the hospital diabetic ketoacidosis protocol. Overnight, the patient's condition deteriorated with worsening shock, worsening mental status with lethargy, and persistent tachypnea, leading to intubation and initiation of supportive mechanical ventilation. Upon reassessment in the morning, the patient was on norepinephrine and vasopressin drips to maintain a mean arterial pressure of 65 mmHg in addition to IV fluids per the DKA protocol. He had significant tenderness to palpation of the abdomen when examination was performed during the spontaneous awakening trial.

**Figure 2:**
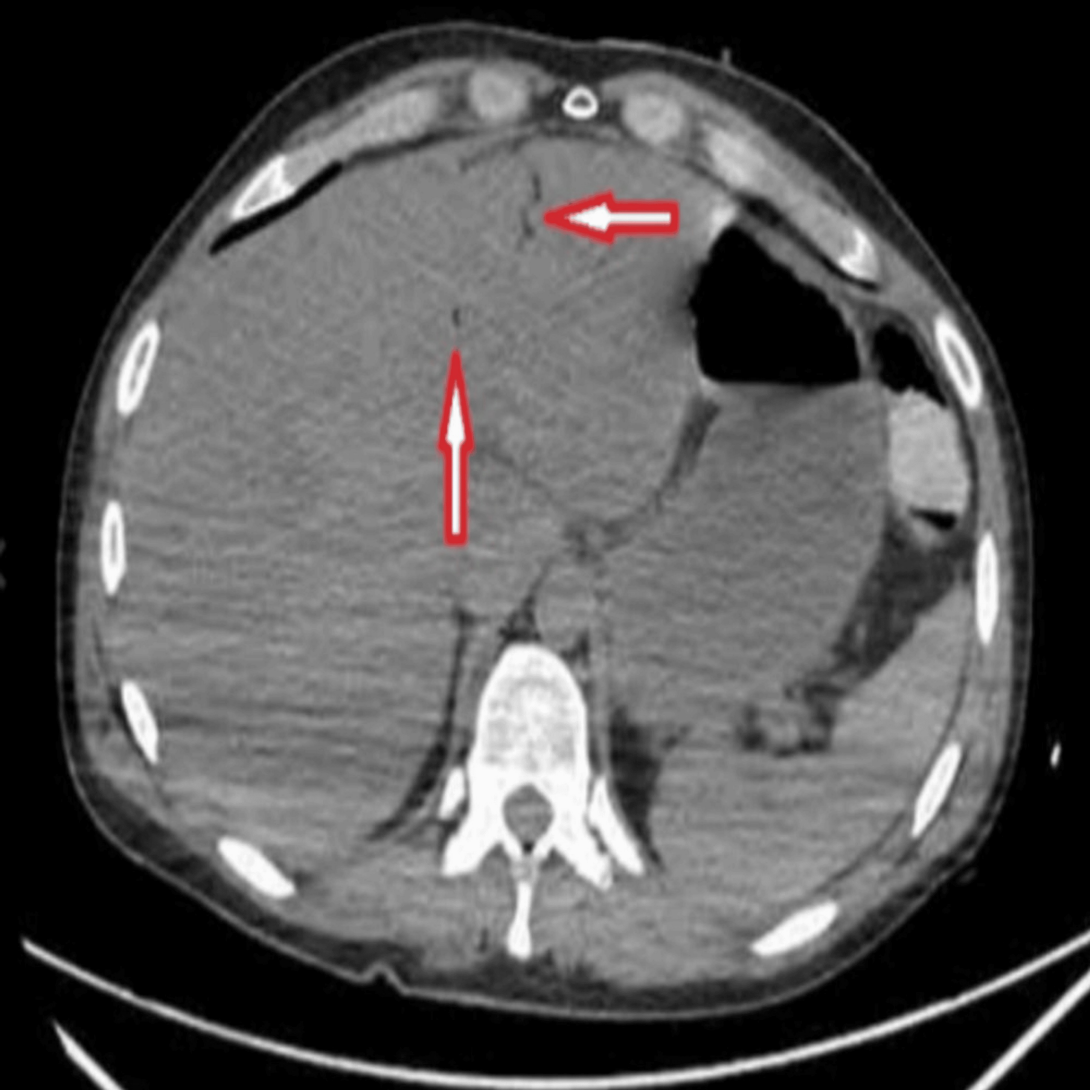
CT abdomen and pelvis, axial (transverse) section, showing portal venous gas (arrow).

**Figure 3:**
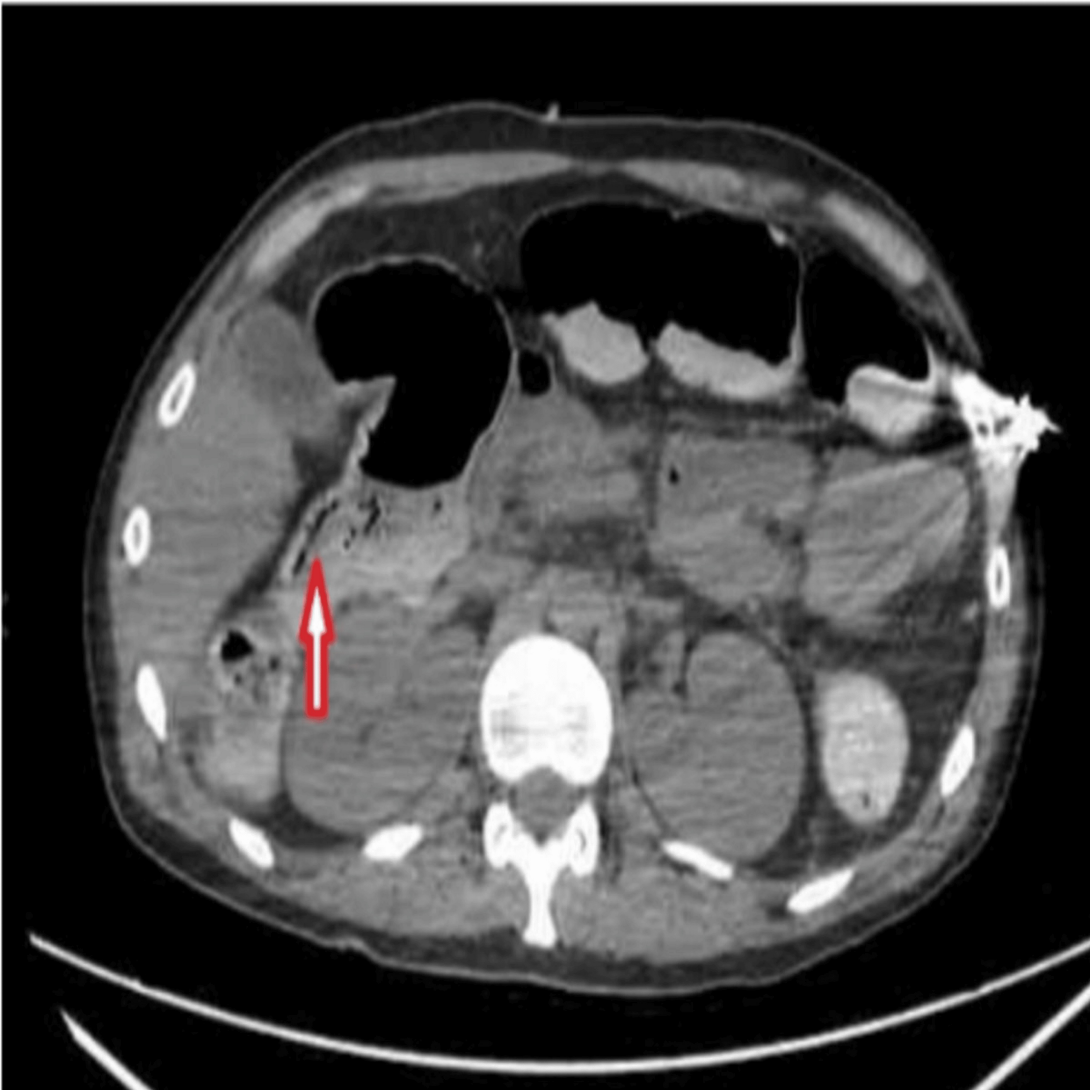
CT abdomen and pelvis, axial (transverse) section, showing pneumatosis intestinalis (arrow).

A computerized tomography (CT) scan of the abdomen and pelvis was obtained and showed portal venous gas (Figure 2) and pneumatosis intestinalis (Figure 3). General surgery was consulted immediately, while further infusion of two liters of crystalloids was administered, and the patient underwent emergent exploratory laparotomy. Extensive ischemic necrosis was noted in the terminal ileum, the right colon, and the appendix. Resection of the terminal ileum and right hemicolectomy was performed. A 26 cm segment of the right colon with attached appendix and 48.5 cm of terminal ileum was removed. The bowel was left in discontinuity, and an ABThera wound VAC was placed. Forty-eight hours later, second-look laparotomy showed no further signs of ischemia; the entirety of the small bowel and large bowel was well perfused. Abdominal washout and ileo-transverse colon anastomosis were performed with abdominal closure. Our patient continued to improve; he was liberated from mechanical ventilation and transferred to the medical floor before being discharged home a few days later.

## Discussion

During severe hypovolemia, a physiological mechanism to preserve perfusion of vital organs by decreasing mesenteric perfusion exists. Prolonged hypoperfusion due to any reason can result in mesenteric vasoconstriction caused by sympathetic overdrive in the peripheries of mesenteric vessels [4]. Disturbance in supply and demand due to persistent mesenteric vasoconstriction makes the intestine, especially the superficial mucosa, vulnerable to ischemic injury [5]. Damage to tissue in NOMI starts as mesenteric vasospasm and can be exacerbated by blood flow restoration, as occurs during DKA fluid treatment, due to ischemia/reperfusion injury [6].

NOMI is a rare complication of DKA in adult patients, with most reported cases involving patients older than 50 years [7]. Older age is often associated with atherosclerotic disease of the mesenteric circulation, predisposing patients to ischemia in low-flow states. Our patient, however, was a young adult male presenting with DKA as the first manifestation of previously undiagnosed secondary diabetes mellitus. This was supported by an elevated HbA1c of 18.4%, indicating chronic hyperglycemia, and a negative GAD antibody test, ruling out type 1 diabetes. Given his history of alcohol-induced pancreatitis, secondary diabetes was the most probable diagnosis. Infectious triggers such as influenza can precipitate DKA by increasing insulin resistance and metabolic demands, tipping the balance in patients with underlying glucose metabolism abnormalities.

Despite adequate treatment of DKA, persistent abdominal pain, hypotension, and rising lactic acid levels raised suspicion for ischemia. Abdominal CT angiogram remains the investigation of choice for diagnosing acute mesenteric ischemia with high accuracy [8]. The presence of portal venous gas and pneumatosis intestinalis on imaging was diagnostic of bowel wall ischemia.

Clinicians should maintain vigilance for complications of DKA beyond the usual triggers, including electrolyte disturbances, acute kidney injury, cerebral edema, hypovolemic shock, and mesenteric ischemia. This case underscores the importance of recognizing secondary diabetes and infection as factors precipitating DKA in patients without a prior diabetes diagnosis. Early identification of these contributors can facilitate prompt management and reduce morbidity.

We also highlight the critical role of volume resuscitation over excessive vasopressor use in isolated DKA cases where hypovolemia is the main driver of shock. These patients typically have increased sympathetic tone and vasoconstriction, and overuse of vasopressors without adequate volume replacement can exacerbate ischemic injuries.

Volume losses can vary widely, and assessment of volume status should be ongoing. In our patient, immediate surgical intervention alongside aggressive fluid resuscitation led to stabilization and recovery. It has been reported that initiating treatment within 24 hours improves survival rates significantly compared to delayed intervention [9].

## Conclusions

Hypovolemia in DKA can lead to hypoperfusion of non-occlusive mesenteric ischemia, a life-threatening complication. Adequate volume resuscitation is a cornerstone of treatment. Early intervention in diagnosing and managing ischemia can lead to a decrease in morbidity and mortality and improve survival.
